# Accuracy of MRI and X‐Ray Measurement of Displacement Distance of Humeral Lateral Condyle Fractures

**DOI:** 10.1111/os.13116

**Published:** 2021-09-19

**Authors:** Lin Guo, Xiaofeng Li, Zhi Wang, Shan Zhu

**Affiliations:** ^1^ Tianjin Hospital Tianjin China

**Keywords:** Elbow, Humeral lateral condyle fracture, Magnetic resonance imaging, X‐ray

## Abstract

**Objective:**

To investigate the accuracy of X‐ray and magnetic resonance imaging (MRI) measurements in evaluating the displacement of humeral lateral condyle fracture (HLCF) in different positions of the forearm based on human cadaveric HLCF models.

**Methods:**

Three human cadaveric elbow HLCF fracture models were successfully established. The wrist joint was fixed, and the forearm was rotated forward along the mid‐axis. The maximum distance between the two segments of the lateral fracture gap was defined as LFS (lateral fracture space) distance, and the maximum distance between the two segments of the fracture gap at the anterior and posterior margins of the fracture model was defined as PFS (posterior fracture space). The LFS and PFS distances of the human cadaveric elbow HLCF fracture models were measured during forearm rotation at 0º, 45º, 90º, and 135º rotation using a Capture Motion System (CMS), positive and lateral elbow X‐ray, coronal and sagittal MRI scans, respectively, and the CMS measurements were considered as the true fracture gap distances. The values obtained by CMS, X‐ray, and MRI measurements for both LPS and PFS distances in the HLCF fracture model at each position during rotation were recorded. The LFS and PFS distances were measured by two independent orthopaedic and joint imaging physicians. The data were measured three times by each physician, and the final values were the average of the two measurements. The outcomes were determined by whether a statistical difference exists in the LFS and PFS among the CMS, X‐ray, and MRI groups.

**Results:**

The interobserver agreement tests between the two observers showed good agreement in the measurements. A multiple sample ANOVA showed statistical differences in the LFS distances of HLCF measured at 0º, 45º, 90º, and 135º rotated by three radiographic measurements (*P* < 0.05). The LFS distances obtained by MRI and CMS measurements were greater than those obtained by X‐ray measurement in all positions using the LSD test (*P* < 0.05), and no statistical difference was found between the CMS and MRI methods in each position (*P* > 0.05). The same results were observed in terms of PFS values obtained by CMS, X‐ray, and MRI measurements at 0º, 45º, 90º, and 135º pronation. It was statistically different among the three groups as shown by multiple sample ANOVA (*P* < 0.05). The CMS and MRI measurements were greater than the X‐ray measurements (*P* < 0.05), while no statistical difference was observed between the CMS and MRI measurements (*P* > 0.05).

**Conclusion:**

X‐rays often underestimate the degree of displacement of HLCF fractures; MRI measurements are closer to the true values compared with X‐ray.

## Graphical abstract

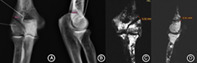

## Introduction

Humeral lateral condyle fracture (HLCF) is the most common type of elbow fracture in children, accounting for 12%–20% of all elbow fractures in children, and second only to supracondylar fractures in children. The incidence of HLCF is estimated at 1.6/1000 per year^1^, ^2^, ^3^. HLCF is mostly an intra‐articular fracture and requires a clear diagnosis and accurate determination of the degree of fracture displacement at the early stage of the fracture, so that clinical classification and selection of treatment modality can be made according to the fracture stability and degree of fracture block displacement and separation. The surgical treatment criterion for HLCF is fracture displacement over 2 mm, either closed reduction with percutaneous internal fixation or incision internal fixation^4^, ^5^. In children with HLCF without a displacement or with a fracture displacement of less than 2 mm, external fixation can be performed with a repositioned plaster rest^6^.

However, it is still common to underdiagnose children with HLCF without significant displacement or to underestimate the severity of HLCF fractures in children and to develop secondary fracture displacement during conservative treatment, resulting in severe late complications such as nonunion, delayed healing, malunion, and ulnar neuritis, affecting the growth and development of elbow joint in children and leading to joint flexion and extension dysfunction or even disability. At present, radiographs are still the primary method for determining HLCF in children, and they are used to identify the specific type of fracture, estimate the degree of fracture separation and displacement, and determine the treatment accordingly^7^. As the humeral tuberosity, lateral condyle, and distal humeral articular surface of the elbow joint in children are largely composed of cartilage tissues and X‐rays can pass through those tissues, many scholars have suggested that X‐rays are not sufficient to assess the extent of fracture displacement in children with HLCF, as the X‐ray measurements that do not show cartilage may lead to missed diagnoses or underestimations of the severity of the fracture. Therefore, accurate diagnosis and assessment of HLCF in children has been a difficult problem for orthopaedic pediatricians^8^. Song *et al*. proposed the importance of internal oblique radiographs of the elbow joint in determining the stability of HLCF without significant displacement^9^. In China and abroad, various tests such as high‐resolution ultrasound, arthrography, and even diagnostic arthroscopy have been attempted to accurately assess the stability of HLCF fractures and the degree of fracture displacement. However, each examination has its own limitations, and inconsistent results have been reported^10^, ^11^, ^12^. Few studies have investigated the accuracy of X‐ray measurements to determine the distance between displaced HLCF fracture gaps at different angles of forearm rotation, and no studies have been reported on the accuracy of magnetic resonance imaging (MRI) to determine the change in displaced HLCF fracture gaps during forearm rotation.

In this study, we established a cadaveric model of HLCF and measured the changes in the lateral/posterior fracture space of the HLCF fracture model during the rotational movement of forearm on different angular positions using three methods: motion capture system, X‐ray, and MRI, using the Capture Motion System (CMS) measurements as the standard. The objectives of this study were as follows: (i) to determine whether X‐ray measurements can correctly assess the distance of HLCF fracture displacement; (ii) the accuracy of MRI in measuring the distance of HLCF fracture gap displacement; and (iii) whether MRI is superior to plain X‐ray measurements in assessing the degree of HLCF fracture displacement in the positive/lateral position of elbow joint.

## Materials and Methods

### Experiment Specimens

The subjects of this trial were three human cadaveric elbow joints that were successfully established as the HLCF fracture models. The cadaveric elbow joints were collected from the anatomy teaching and research department of Tianjin Medical University and the anatomy laboratory of Tianjin Orthopaedic Institute, including: one male, 65 years old, left elbow joint; one male, 44 years old, right elbow joint; and one female, 57 years old, left elbow joint. The specimens were autopsied and frozen cadaveric forearms. The skin, muscle, blood vessels, nerves, and fascial tissue of the elbow joint and forearm and humerus had been removed, and the specimens were frozen and preserved at −25°C. The HLCF fracture model was created through a posterior lateral approach. Care should be taken during specimen preparation to keep the radial collateral ligament of the lateral epicondyle of the humerus, all extensor resistances, and the elbow capsule intact.

### Main Materials and Experimental Equipment

The humerus of elbow joint was fixed without relative movement during the experiment. The proximal end of humerus was threaded with a 2.0 mm kerf pin and fixed in the device to ensure that no rotational movement or inversion/eversion of the humerus occurs during forearm rotation. A special stopper was placed on the hand, which both elevates the wrist and keeps it leveled with the forearm to avoid flexion/extension of the wrist joint, and also allows the stopper to be used to hold the medial and lateral edges of the hand to maintain the stability of the hand and forearm after rotation to a certain angle. For anteroposterior elbow X‐rays (Discovery XR656, GE, USA), the following parameters were used: tube voltage 56 kV, tube current 5 mAs; for lateral elbow images: tube voltage 60 kV, tube current 5 mAs. The standard lateral elbow images require that the medial and lateral condyles of the humerus overlap on the image.

The MRI scanner was equipped with a 3.0T MR (Discovery MR 750;GE;USA) and an eight‐channel phased‐array shoulder coil to scan the distal humerus in each rotational position and acquire two sequences, coronal 3D‐FSPGR T1‐weighted images (TR = 7.8 ms; TE = 3.8 ms) and sagittal T2‐weighted images (TR = 3000 ms; TE = 90.7 ms). Both sequences required a layer thickness of 3.0 mm and two excitation times.

The realistic fracture displacement of the HLCF fracture model was measured using a CMS (Optotrak 3020, NDI, Waterloo, Canada). The spatial three‐dimensional displacement of HLCF fragment relative to the distal humerus during forearm rotation was accurately measured, and the change in displacement distance between the lateral fracture space (LFS) and the posterior fracture space (PFS) was calculated using computer technology and image processing by varying the coordinate positions of spatially marked points located at the ends of fracture gap. At a distance of 2.25 m from the object being probed, the Optotrak 3020 motion capture system has an accuracy of 0.1 mm and a resolution of 0.01 mm. Two motion markers are positioned on each side of the humeral epicondyle fracture end (i.e. the lateral humeral condyle fracture fragment and the distal humerus) by using 2.0 Gram pins, ensuring that the marker points are firmly fixed and do not wobble during movement. Three pairs of positioning points are fitted to each motion marker to represent the spatial X, Y, and Z axes, and the position of each marker is recorded, giving each marker a spatial positioning and pairing to determine the spatial position of fracture surface. During the rotation of the forearm, the change in the spatial position of fracture ends was recorded using a motion capture system.

### Measurement Methods

#### Lateral Fracture Space (LFS) Distance

**Fig. 1:**
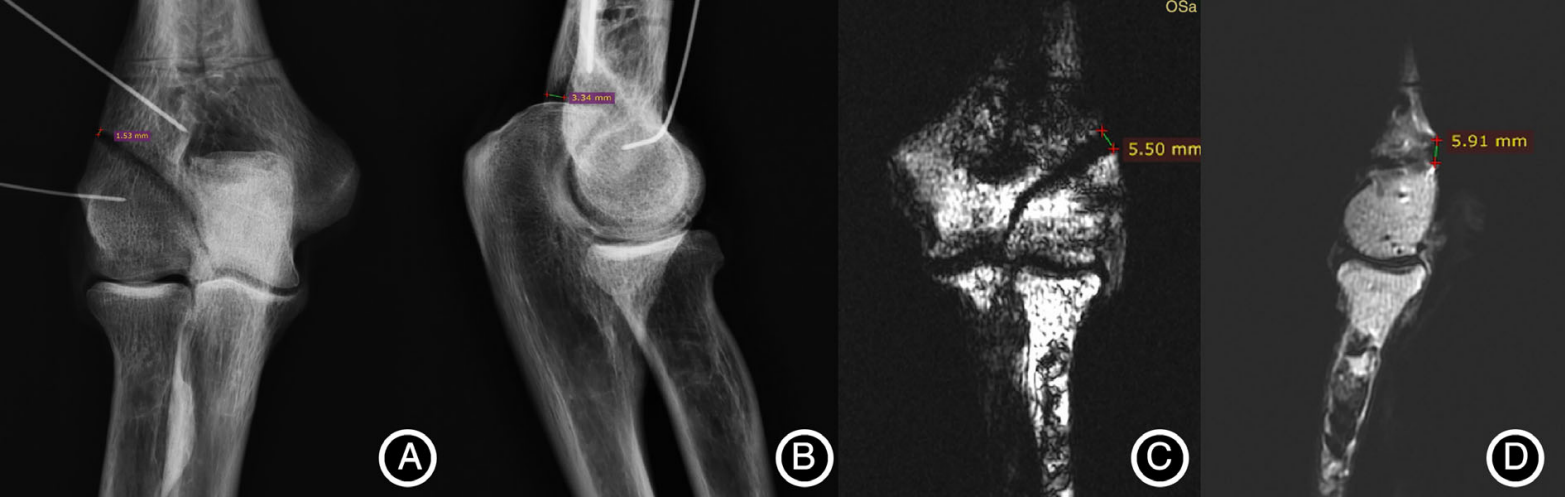
The measurement of the LFS/PFS distance of the fracture model on the X‐ray and MRI of the elbow joint. (A) Measurement of LFS distance on anterior–posterior radiograph. (B) Measurement of PFS distance on lateral elbow radiographs. (C) Measurement of LFS distance on coronal T1WI of the elbow MRI. (D) Measurement of PFS distance on sagittal MRI T2WI of the elbow joint.

The maximum distance between the two broken ends of the most lateral fracture gap, the LFS distance, was measured from the orthogonal X‐ray as well as MRI coronal images of elbow joint in the HLCF fracture model (Fig. 1A and C). Owing to the HLCF fracture mechanism, the fracture line mostly travels from the epicondylar edge of the humerus towards the medial inferior humeral intercondylar glide, and the fracture fragment is pulled by the extensor tendon of the humeral epicondyle. The treatment plan is usually based on the measurement of the size of LFS in HLCF in clinical practice.

#### Posterior Fracture Space (PFS) Distance

The maximum distance between the two segments of the fracture gap at the anterior and posterior margins of the HLCF fracture model, the PFS, was measured on the lateral X‐ray and MRI sagittal images of elbow joint (Fig. 1B and D). Fracture displacement is often observed in the sagittal plane, too. Sometimes the fracture displacement in the sagittal plane exceeds that in the coronal plane. However, due to the shape characteristics of the HLCF fracture line, it is usually not easy to observe the anteroposterior displacement of the fracture in the sagittal plane compared with the coronal plane. If the fracture displacement distance in the sagittal plane or the coronal plane reaches the surgical indication, surgery should be taken. Measurement of the LFS alone does not fully determine the extent of fracture displacement.

### Measurement of Fracture Gap Displacement During Forearm Rotation

The cadaveric HLCF model was placed on the experimental manipulation table, and the proximal humerus was securely fixed to prevent the rotation of humerus after the fracture ends were fitted with motion marker points. The specimen was initially positioned at 0º of forearm rotation anteriorly (palm side of the hand facing upwards). The forearm was rotated sequentially at 0°, 45°, 90°, and 135° (by performing an anterior rotation movement).

In order to ensure that the forearm rotation angle is accurate, the rotation angle of the forearm needs to be determined after measurement by a protractor, and the measurement accuracy of the rotation angle is up to 1º. For the purpose of the consistency of the measurement data, the measurement of the same body position by the three measurement methods needs to be completed in 1 day. During the transportation from one examination equipment to another, the fracture model should be firmly fixed by clips on the hand, elbow, and proximal humerus to prevent the forearm from moving.

**Fig. 2:**
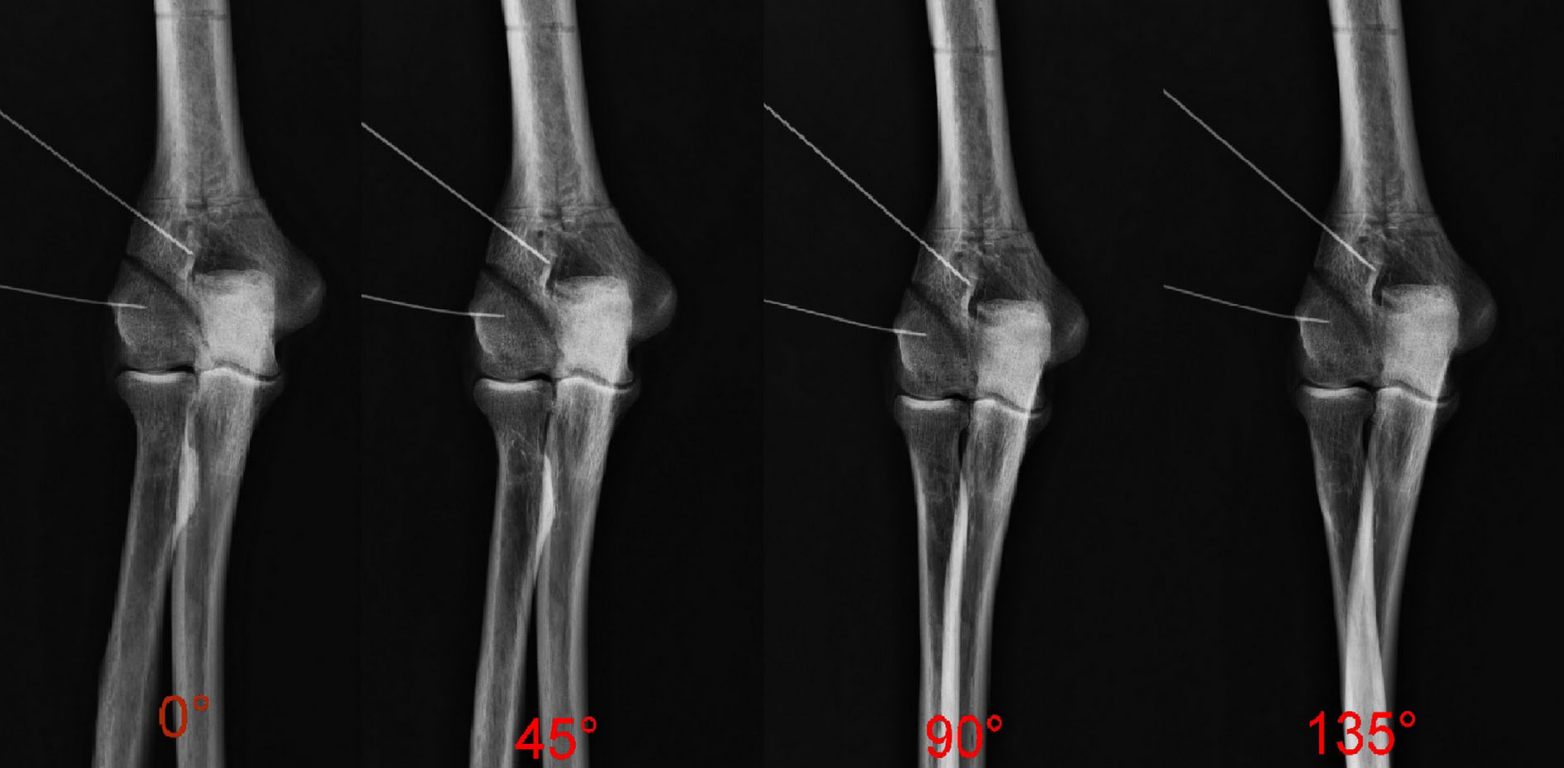
Anteroposterior X‐ray of the elbow joint after rotation of the forearm (from posterior to anterior rotation) at 0°, 45°, 90°, and 135°, with the LFS distance of the fracture model measured and recorded in each position.

**Fig. 3:**
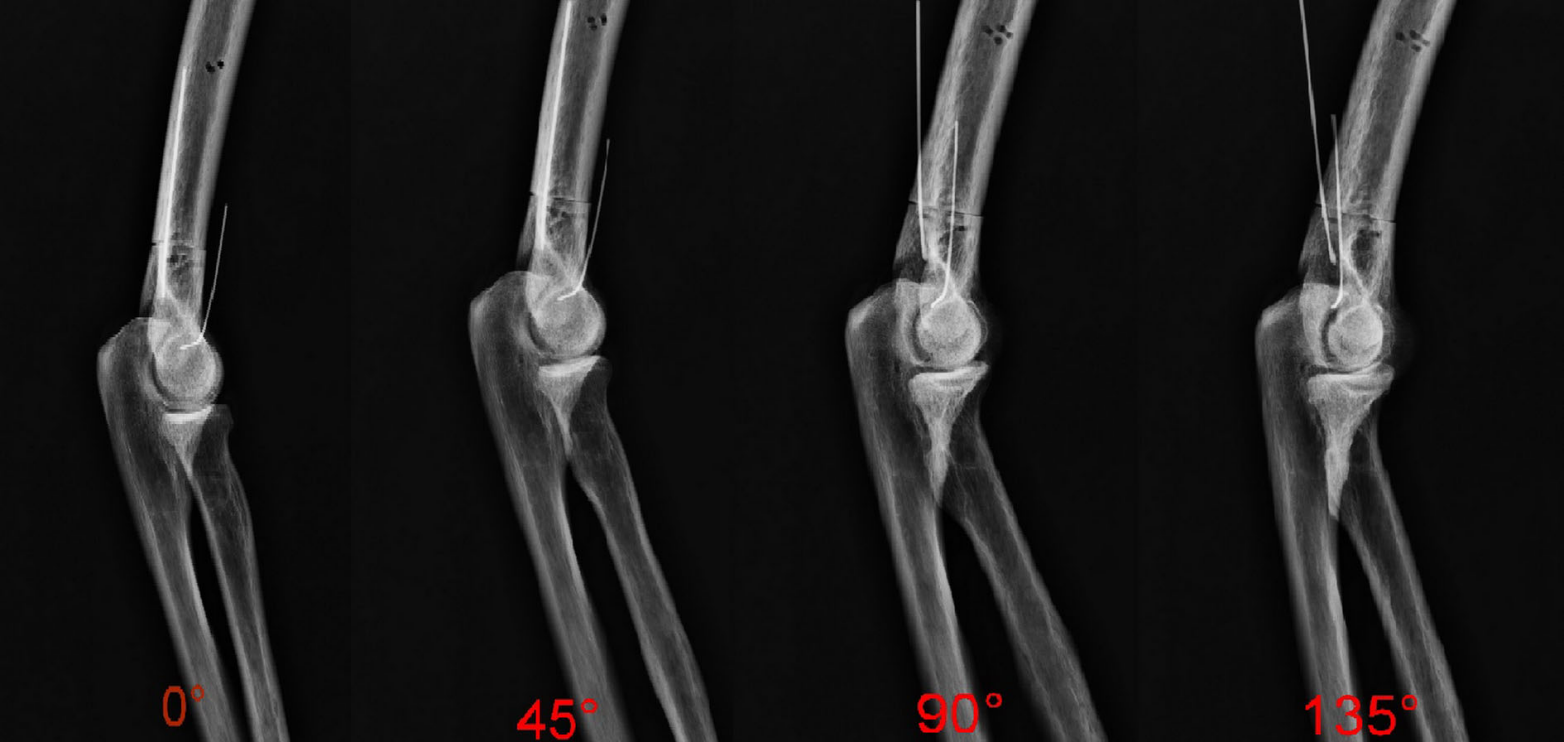
Lateral X‐ray of the elbow joint after rotation of the forearm (from posterior to anterior rotation) at 0°, 45°, 90°, and 135°, with PFS distance measured and recorded in each position.

**Fig. 4:**
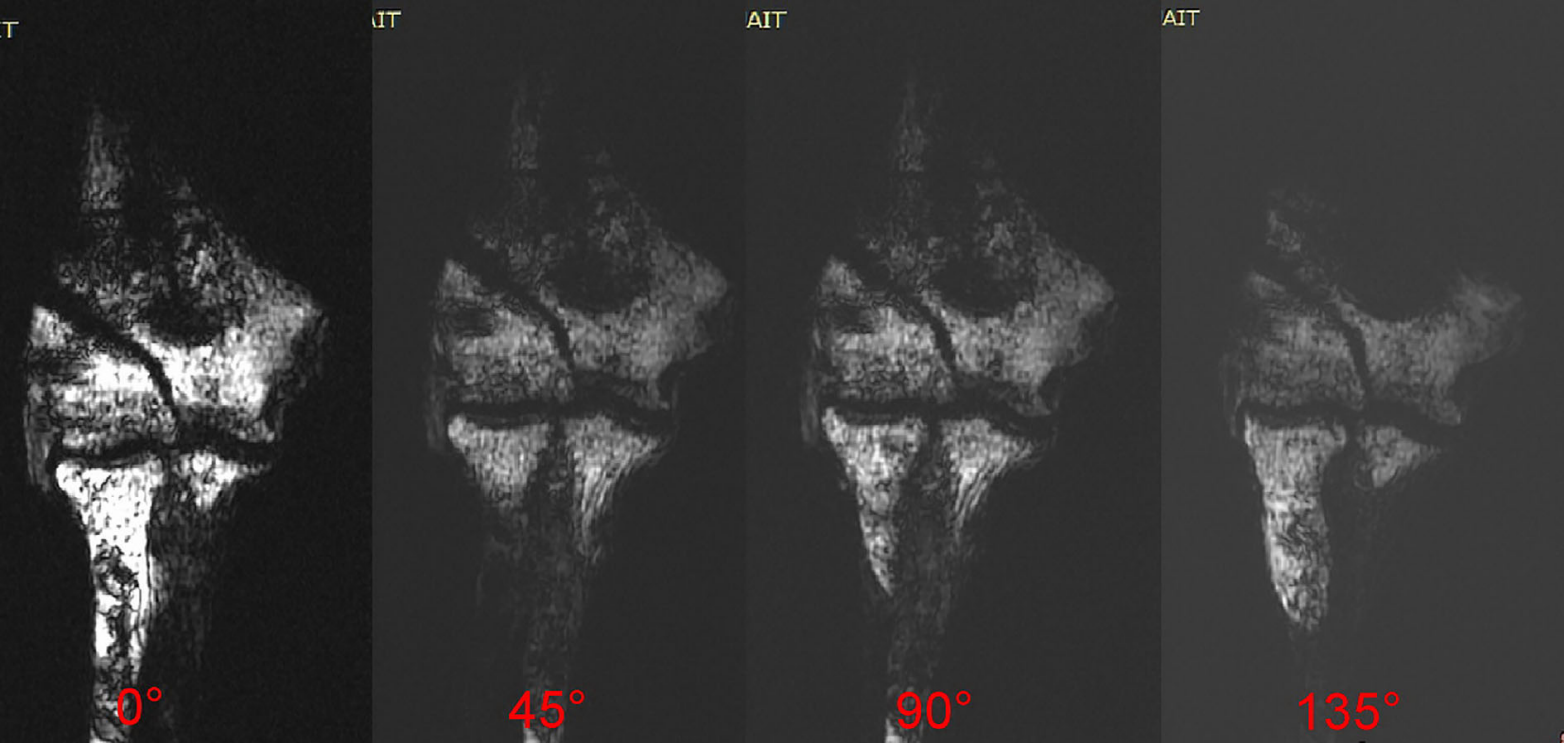
Scanning of the distal humeral coronal 3D‐FSPGR T1WI after rotation of the forearm (from posterior to anterior rotation) at 0°, 45°, 90°, and 135°, with measurement and recording of the LFS distance of the fracture model in each position.

**Fig. 5:**
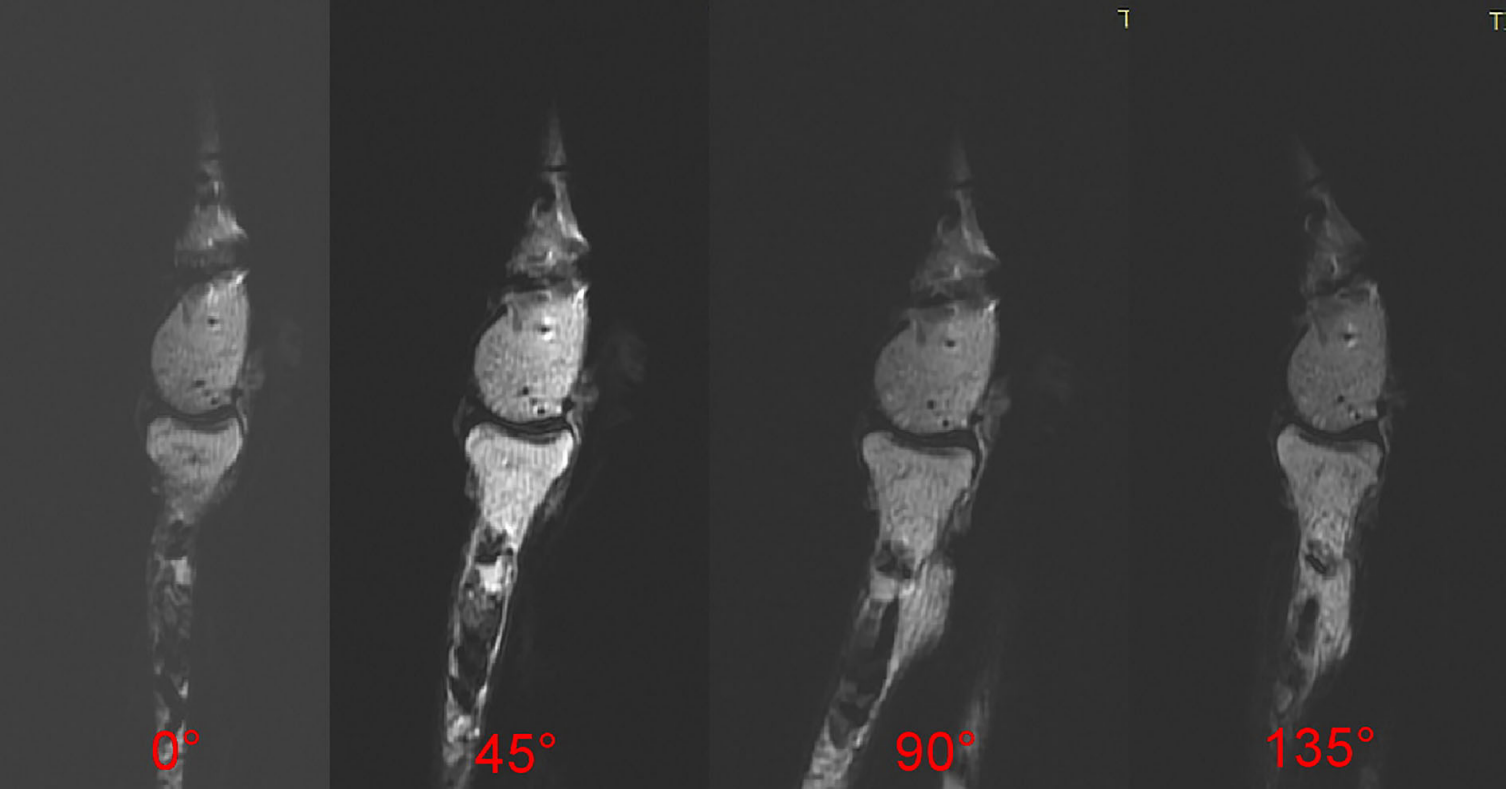
T2WI of the distal humerus in sagittal position after forearm rotation (from posterior to anterior rotation) at 0°, 45°, 90°, and 135°, with PFS distances measured and recorded for each body position.

The LFS and PFS of the HLCF were measured using CMS, X‐ray, and MRI in each body position at rest. The values obtained from the motion capture system were the true displacement distances. Two elbow radiographs were taken in each position, with the LFS distance measured on anterior–posterior radiographs (Fig. 2) and the PFS distance measured on lateral radiographs (Fig. 3). The distal humerus was scanned using MRI, layer‐by‐layer to identify the median coronal plane with the largest fracture gap, and the LFS and PFS distances were measured from the median coronal images (Figs 4 and 5).

### Data Analysis

The LFS and PFS distances were measured by two independent orthopaedic and joint imaging physicians on the MRI coronal and sagittal images in each body position during the forearm rotation on a GE image processing workstation (Workstation AW46.2, GE, Milwaukee, USA); and on the PASC system (SYNAPSE, DICOM version 3.0, FUJIFILM Medical System, Stamford, USA). The LFS and PFS distances were measured in each position during forearm rotation on anterior–posterior and lateral X‐rays of the elbow joint. The data were measured three times by each physician, and the final values were the average of the two measurements. Using CMS, the change in the spatial position of each digitally marked point corresponding to each body position was measured and recorded in real time. Shift distances were recorded and calculated using NDI's First Principles software (NDI, Waterloo, Canada).

### Statistical Analysis

The data obtained from this study were statistically analyzed using the SPSS 20.0 (SPSS Inc., USA) software package. An intraclass correlation coefficient (ICC) test was performed to evaluate interobserver agreement between two observers. The LFS and PFS distances of HLCF were measured using the motion capture system, X‐ray, and MRI, and the measurement data were expressed as mean ± standard deviation. Analysis of variance (ANOVA) was used to compare the measurement data between the three groups. First, the data of the three groups were tested by normal distribution test and chi‐square Levene test to determine whether the statistical data of the three groups conformed to normal distribution and the consistency of the overall variance between the samples, and then a one‐way ANOVA test was used to determine whether the means of the three groups were statistically different. A least significant difference multiple comparison method was used to determine whether a statistical difference existed between the two comparisons of CMS, X‐ray, and MRI groups. The test level was 0.05 for both sides, and *P* < 0.05 was considered as statistically significant.

## Results

### Interobserver Agreement Test for Data

The LFS and PFS of HLCF fracture model were measured from the X‐ray anterior/lateral and MRI coronal/sagittal views of the elbow, respectively, and the interobserver agreement tests between the two observers showed good agreement in the measurements. The ICC values for the LPS obtained from X‐ray and MRI measurements were 0.915 and 0.934, respectively, while the ICC values for the PFS obtained from X‐ray and MRI measurements were 0.865 and 0.901, respectively.

### Measurements of Lateral Fracture Space (LFS) in Humeral Lateral Condyle Fracture (HLCF)

The LFS distances of HLCF measured at 0º, 45º, 90º, and 135º rotation along the mid‐axis of the forearm in each position on CMS, X‐ray, and MRI coronal images were tested for the normal distribution and overall homogeneous variance in all the three groups, and a multiple sample ANOVA showed statistical differences in the means among the three groups (*P* < 0.05) (Table 1).

**TABLE 1:** LFS values obtained from CMS, X‐ray, and MRI measurements in each position

Method	Pronation 0°	Pronation 45°	Pronation 90°	Pronation 135°
CMS	4.39 ± 0.56	5.45 ± 0.87	5.05 ± 0.57	6.24 ± 0.98
X‐ray	1.39 ± 0.06	1.47 ± 0.09	1.66 ± 0.11	1.59 ± 0.33
MRI	3.97 ± 0.65	5.24 ± 1.04	4.77 ± 0.32	5.76 ± 0.67
*F* value	31.947	24.299	72.752	39.002
*P*‐value	0.001	0.001	0.000	0.000

CMS, Capture Motion System; LFS, lateral fracture space.

The LFS distances obtained from MRI and CMS measurements were greater than those obtained from X‐ray measurements in all positions (Table 1). The LFS values obtained from the X‐ray measurements did not vary significantly with forearm rotation, with values varying by less than 1 mm (from 0.14 to 0.67 mm) in all positions, whereas the LFS values obtained from MRI and CMS measurements during forearm rotation varied more (MRI: from 1.30 to 4.12 mm; CMS: 1.54 to 4.71 mm) than those obtained from the X‐ray measurements.

The mean LFS values obtained from the three examination methods in each position were analyzed by ANOVA with multiple sample measures, showing a statistical difference among the three groups (*P* < 0.05); the LSD test conducted for comparing the two groups showed no statistical difference in the LFS measurements between the CMS and MRI methods in each position (at 0° pronation of the forearm, *P* = 0.367, while at 45°, 90°, 135° pronation of the forearm, *P* = 0.755, 0.405, 0.445, respectively). Significant statistical differences were obtained between the CMS and X‐ray (*P* < 0.001). The same outcomes were obtained between the MRI and X‐ray (*P* < 0.001).

### Measurements of Posterior Fracture Space in Humeral Lateral Condyle Fracture (HLCF)

The PFS distances for HLCF measured at 0º, 45º, 90º, and 135º rotation along the mid‐axis of the forearm in each position on CMS, X‐ray lateral, and MRI sagittal images, which were tested for the normal distribution and overall homogeneity of variance in the three groups, and a multiple sample ANOVA showed statistical differences in the means between the three groups (*P* < 0.05) (Table 2).

**TABLE 2:** PFS values obtained from CMS, X‐ray, and MRI measurements in each position

Method	Pronation 0°	Pronation 45°	Pronation 90°	Pronation 135°
CMS	6.24 ± 0.73	7.27 ± 0.28	6.78 ± 0.43	8.04 ± 1.50
X‐ray	1.92 ± 0.13	2.71 ± 1.24	2.45 ± 0.51	2.20 ± 0.63
MRI	5.57 ± 0.38	6.96 ± 0.50	6.11 ± 0.55	7.02 ± 1.26
*F* value	70.151	35.996	64.368	43.535
*P* value	0.000	0.000	0.000	0.000

CMS, Capture Motion System; PFS, posterior fracture space.

The values of PFS obtained using the three examination methods for HLCF in each position were statistically different among the groups with multiple sample measures (*P* < 0.05); and later LSD multiple group comparison tests between the pairwise groups showed no statistical difference between PFS measured by CMS and MRI sagittal images in each position (at 0° pronation of the forearm, *P* = 0.138, while at 45°, 90°, 135° pronation of the forearm, *P* = 0.620, 0.156, 0.179, respectively). A statistically significant difference (*P* < 0.001) was observed between CMS and X‐ray. A statistically significant difference (*P* < 0.001) was observed between MRI and X‐ray method.

## Discussion

HLCF is the most common type of intra‐articular fracture in children and requires a clear diagnosis and accurate determination of the degree of fracture displacement at the early stage of fracture to allow clinical staging and treatment selection based on the fracture stability and degree of fracture displacement separation. At present, the diagnosis of HLCF in children and the determination of degree of fracture displacement are still based on conventional X‐ray examination^7^.

X‐ray examination is widely used in the diagnosis of HLCF in children because it is convenient, inexpensive, and widely available, and fracture staging based on X‐ray findings is also relatively simple and practical, and has been used by many clinical practitioners. After making clinical decisions based on X‐ray measurements, some children with HLCF fractures still have a poor prognosis in practice, especially those with an initial X‐ray diagnosis of non‐displaced HLCF who develop a secondary fracture displacement during conservative treatment. In many of these children, failure to detect the fracture misalignment in time or underestimation of the extent of fracture displacement separation delays surgery, resulting in severe complications such as delayed fracture healing or nonunion. Therefore, the issue of accuracy^4^, ^13^, ^14^ of X‐ray assessment of HLCF in children has become an important research topic and a difficulty in the diagnosis and treatment of HLCF in children^8^.

In this study, the changes in fracture gap during forearm rotation were simulated and measured using a human cadaveric elbow HLCF fracture model. Fracture displacement was measured using CMS, X‐ray, and MRI methods at 0º, 45º, 90º, and 135º forearm pronation during the rotation about the central axis of the forearm, while the projected position of the forearm up to 135º pronation was close to the position of the HLCF patient present in the emergency room for internal oblique radiographs. The results of this study show that the LFS obtained from MRI measurements in the coronal position was closer to the true LFS obtained from CMS measurements. However, the PFS obtained from MRI measurements in the sagittal position was closer to the true PFS obtained from CMS measurements, while the LFS and PFS values obtained from the X‐ray measurements at all positions during rotation were both significantly different from those obtained from CMS and MRI measurements. Moreover, both the LFS and PFS values obtained from the X‐ray measurements were significantly smaller than those obtained from MRI and CMS measurements. The results of this study show that the measurement of HLCF fracture displacement distance on the X‐ray is not accurate.

Both the LFS and PFS values obtained from the X‐ray measurements during the forearm rotation varied slightly, both LFS and PFS varying essentially less than 1 mm with forearm rotation, whereas the values obtained from MRI measurements were close to the true fracture displacement distance of CMS. The true LFS values obtained from CMS measurements ranged from 1.5 to 4.7 mm. The PFS values ranged from 1.6 to 4.5 mm in all positions. However, the LFS values obtained by MRI imaging ranged from 1.3 to 4.1 mm, and the PFS values ranged from 1.4 to 4.9 mm. The statistical results also confirmed that the LFS and PFS values obtained from MRI measurements in all positions were not statistically different from those obtained from CMS measurements. The trend in HLCF fracture gap distance obtained from the three examinations showed that the LFS and PFS were significantly greater for both CMS and MRI than for X‐ray measurements, and that the CMS and MRI measurements were a good fit. It can be therefore concluded that the LFS and PFS values obtained from the X‐ray measurements are both smaller than the true fracture displacement distance and smaller than those obtained from the MRI measurements, regardless of the rotational position of the forearm. This indicates that the fracture displacement distance obtained from the X‐ray measurements tends to underestimate the true extent of fracture displacement. Currently, a fracture displacement of more than 2.0 mm is usually considered an indication for surgical treatment of HLCF in children, and HLCF with a fracture displacement of less than 2.0 mm can be treated conservatively with external fixation^5^, ^15^, ^16^. For such a small distance, failure to accurately assess the degree of fracture displacement by X‐ray measurements may severely affect the physician's judgment of the fracture itself, leading to incorrect treatment choices with irreversible consequences. The results of this study show that X‐ray assessment of the distance of fracture displacement in HLCF should be used more carefully as a basis for treatment selection, and that clinical errors resulting from the X‐ray underestimation of the degree of fracture displacement should be avoided.

This study also found that the MRI method is more accurate than the X‐ray method in measuring the displacement distance of HLCF fractures. This is probably because the two imaging methods are different in principle: the X‐ray method is not conducive to the observation of fracture gap and does not facilitate the accurate measurement of gap displacement width due to the overlapping of image structures, whereas the MRI method is a multidirectional, multiangle tomographic imaging that can be adjusted according to the needs of the measurement, and can clearly show the alignment of the fracture line and determine the fracture gap displacement at the largest level when measuring layer by layer. The MRI method is therefore significantly better than the plain X‐ray method in assessing the extent of fracture displacement in HLCF. The use of MRI method for fracture typing and fracture separation gap measurement in children with HLCF has the potential to change treatment choices, thus compensating for the inadequacy of plain X‐ray method and avoiding the adverse consequences of poor clinical decision solely based on the X‐ray assessment of degree of fracture displacement.

When comparing the LFS and PFS values obtained from the three methods, we find that the overall PFS is higher than the LPS, but there are also subtle differences between the measurements. The LFS obtained from both CMS and MRI measurements is significantly smaller than the PFS, with the largest difference between the LFS and PFS obtained from CMS measurement occurring at 135º pronation, with a difference of 4.5 mm, and the largest difference between the two was obtained from MRI measurement also occurring at this location, with a difference of 4.2 mm. This indicates that it is difficult to accurately display and measure the PFS on X‐ray lateralization, and that the PFS underestimates displacement to a greater extent than the LFS. In practice, we have also found that it is not as easy to show the posterior margin gap in the lateral X‐ray position as it is in the anterior–posterior X‐ray position, especially when the X‐ray projection is at an angle to the fracture gap, which is not conducive to showing the fracture line.

### Limitations of This Study

However, there are still many shortcomings of this study: (i) The specimens used in this study for the preparation of cadaveric elbow joint HLCF model were adult elbow joints. The anatomical and structural characteristics of the adult elbow joint are significantly different from those of children who are still growing and developing. (ii) The number of specimens used in this study is limited. Also the skeletal differences between cadaveric specimens due to gender, age, and growth conditions were significant, and the individual anatomical differences and geometry of these elbow joints may also affect the final results. Therefore, our results also need to be confirmed by further studies with larger sample sizes.

### Conclusion

By studying the changes in fracture gap in a human cadaveric elbow HLCF fracture model under forearm rotation, it was found that the fracture gap displacement distance obtained from X‐ray measurement was significantly smaller than the true displacement distance obtained from CMS measurement. Thus, X‐ray plain radiographs tend to underestimate the degree of displacement of HLCF fractures. The CMS and MRI measurements were similar in all positions, and the MRI measurements were also higher than the X‐ray measurements, making MRI superior to X‐ray plain films in assessing the degree of displacement of HLCF fractures. Therefore, X‐ray measurements alone may not be accurate in determining the distance of fracture displacement as an indication for surgery. Some patients who require surgery are treated conservatively due to the possibility of underestimation of the fracture displacement distance using X‐ray, which inevitably increases the chance of secondary fracture displacement in these patients and may lead to severe complications such as nonunion, delayed healing, or malunion.
